# Identification of cellular enhancing and restricting factors of dengue virus egress

**Published:** 2011-01-10

**Authors:** Pei Gang Wang, Mateusz Kudelko, Kevin Kwok, Roberto Bruzzone, Beatrice Nal

**Affiliations:** 1 HKU–Pasteur Research Centre, Hong Kong, Hong Kong SAR

## 

Dengue has emerged as the most important life-threatening illness in the world, especially in Asian countries around Hong Kong, where the incidence of dengue hemorrhagic fever (DHF) is much greater than other continents. However, little is known about molecular and cellular processes sustaining egress of dengue virus in the host cell.

To better understand the viral and cellular determinants of dengue virus egress, we have established a dengue VLP producing stable cell line (HeLa-prME) and demonstrated that dengue VLP was able to mimic the budding and egress process of dengue viruses so that it constitutes a safe and convenient tool for the study of egress of dengue virus. Under the support of RFCID grant, HeLa-prME cells were used to screen a siRNA library that included 122 cellular membrane trafficking genes.

Our screen results revealed that knockdown of ADP-ribosylation factor (ARF) 1 and ARF6 had significant effects on VLP production by HeLa-prME cell. Experiments with other ARF proteins, which were not included in the siRNA library, showed that the ARF4/ARF5 double knockdown could inhibit VLP production but had no effect on secretion of other proteins such as soluble dengue E protein, suggesting the specificity of their involvement in VLP production. Further experiments using real virus showed that the depletion of ARF4/5 by siRNA could significantly reduced the replication of dengue 1 virus, dengue 4 virus and yellow fever virus, confirming the important role of ARF4 and ARF5 for not only dengue viruses but also other flaviviruses.

Our study uncovered the importance of class II ARFs in the egress of dengue virus. Results from this project provided information on mechanism of dengue virus assembly and its dependence on cellular machineries.

